# Psychophysiological Effects of Biographical Interventions in People With Unresponsive Wakefulness Syndrome and Minimally Conscious State

**DOI:** 10.3389/fneur.2022.788588

**Published:** 2022-05-06

**Authors:** Teresa Grimm, Martin Groß, Urs M. Nater, Oliver Summ, Gunter Kreutz

**Affiliations:** ^1^Public Health Department of the City of Oldenburg, Oldenburg, Germany; ^2^Department of Music, Faculty of Linguistics and Cultural Studies, Carl von Ossietzky University, Oldenburg, Germany; ^3^Department of Neurological Intensive Care and Rehabilitation, Evangelisches Krankenhaus Oldenburg, Oldenburg, Germany; ^4^Research Network on Emergency and Intensive Care Medicine Oldenburg, Faculty of Medicine and Health Sciences, Carl von Ossietzky University, Oldenburg, Germany; ^5^Department of Clinical and Health Psychology, Faculty of Psychology, University of Vienna, Vienna, Austria; ^6^Faculty of Medicine and Health Sciences, Carl von Ossietzky University, Oldenburg, Germany

**Keywords:** disorders of consciousness (DOC), unresponsive wakefulness syndrome (UWS), minimally conscious state (MCS), biographical music, biographical language, cortisol, amylase, dehydroepiandrosterone (DHEA)

## Abstract

**Background:**

Various music interventions can evoke favorable behavioral responses or physiological reactions in people with disorders of consciousness (DOC), such as coma, unresponsive wakefulness syndrome (UWS), and minimally conscious state (MCS). However, it appears that no study thus far has investigated the effects of music on the endocrine system of people with DOC.

**Objective:**

This explorative study aimed to investigate the effects of biographical music and biographical language on the physiological and endocrine systems of people with UWS and MCS.

**Method:**

A cohort of 20 people with DOC (10 women, 10 men; age range 19–77) received 20 min of biographical music and biographical language. Before and afterward, they were exposed to silence. Physiological and hormonal measurements were conducted before, during, and after the interventions.

**Results:**

Paired *t*-tests showed a significant decrease of salivary cortisol in the condition with biographical language interventions.

**Conclusion:**

Biographical interventions can modulate reactions in the endocrine system in people with DOC. Further studies are needed to establish whether and how individuals living with DOC show psychoneuroendocrine responses to music and other arts-based interventions.

## Introduction

### Disorders of Consciousness

Unresponsive wakefulness syndrome (UWS) and minimally conscious state (MCS) belong to disorders of consciousness (DOC). Most patients with DOC show signs of severely disturbed awareness (1, 2) and are unable to communicate verbally or gesture intentionally. UWS is also known as a vegetative state [VS; (3)]. People who live with this syndrome show spontaneous eye opening and a sleep-wake rhythm (4). They are able to breathe independently, but are fully dependent on assistance with activities of daily living (5). Moreover, they are unable to communicate verbally or to respond purposefully to commands (5). A considerable number of neurological patients (43%), particularly those with severe visual impairment or blindness, are misdiagnosed with UWS (6).

People with MCS show visual fixation and other physical reactions to external stimuli that are more than simple reflexes (7, 8). MCS can be divided into MCS PLUS (MCS+) and MCS MINUS (MCS–). People with MCS+ are reproducibly able to respond to commands, whereas people in MCS– are unable to do so (8, 9). UWS and MCS are not permanent states as they can change and even improve (10).

Following earlier work on diagnostic procedures and indicators to assess the severity of DOC (11, 12), state-of-the-art diagnostic tools, such as the Coma Recovery Scale Revised [CRS-R; (13, 14)], and measurements of brain functions via imaging methods (15, 16) have been developed to improve the diagnosis of DOC.

### Rehabilitation of People With DOC

Rehabilitation in DOC focuses on enhancing the quality of life and reducing comorbidity. Therefore, treatment strategies, which potentially elicit biographical memories as residual cognitive resources by means of multimodal perceptual stimulation [e.g., (17, 18)], non-verbal modes of communication, and social interaction, are needed (19). Sensory stimulation therapy offers olfactory, gustatory, tactile, kinesthetic, and auditory stimulation to activate patients and potentially provoke interactive responses (17, 18). Exposure to emotionally charged sounds, such as the patient's name (20), the voice of a relative (21), or music (22), has also been shown to have therapeutic value.

### Effects of Music Interventions and Music Therapy for People With DOC

There are various possibilities for the use and adaption of music for people with DOC. A systematic review by Grimm and Kreutz (23) analyzed 22 quantitative studies with 329 participants with non-degenerative DOC and various musical interventions, such as music therapy, biographical music, and music combined with other interventions. The review found that music interventions may evoke favorable behavioral or (neuro-)physiological reactions in people with DOC. However, the methodological quality of the involved studies was heterogeneous. The mean number of participants was 14.95 per study. Some studies used no established diagnostic assessment, and effect sizes were not reported in most studies (23). A recent systematic review (24) included both qualitative and quantitative studies. The quantitative studies that were published since 2018 showed that music interventions led to an increase in brain connectivity (25, 26), a higher activity of the autonomic nervous system (27), or an increase in behavioral responses (28). One study (29) showed no difference in skin conductance (SC) between the music condition and the control conditions (a neutral sound and olfactory stimulation). In some studies, brain activity was higher, when the patient's name was called (30, 31).

Individual music therapy for people with DOC has been shown to lead to better conditions and significant behavioral responses when compared with environmental sounds (32). Additionally, a pilot study by Steinhoff et al. (33) documented active music therapy conducted three times per week for 5 weeks to lead to an increase in brain activity in the frontal areas, the cerebellum, and the hippocampus.

In a study conducted by Sun and Chen (34), preferred music was played for participants with DOC. Half of the 40 participants received preferred music for 4 weeks in the morning, afternoon, and nighttime prior to sleep. The control group received standard care without music intervention. A comparison of the *Glasgow Coma Scale* (GCS) showed a significantly higher score of the music group when compared with the control group. Moreover, the quantitative value of electroencephalography (EEG) differed significantly between the music group and the control group. The δ + θ/α + β value of the music group was decreased after the 4 weeks with music interventions. This may indicate a positive effect of the intervention on the patients' outcome as the slow wave frequency bands δ and θ are associated with a poor outcome in people with DOC, whereas the fast wave frequency bands α and δ are associated with a favorable outcome (35, 36). Puggina and da Silva (37) examined 76 participants who were sedated, comatose, or lived with UWS. A third of the participants listened to biographical music, another third listened to a message by a relative, and the last third listened to silence. During these interventions, heart rate (HR), blood pressure, body temperature, and oxygen saturation (SpO_2_) were measured. Electric activity of the frontalis muscles and a hand extensor muscle was acquired by using electroneurography (ENG), and facial expressions were recorded with a video camera. The results showed that biographical music caused significant changes in the ENG signal. Biographical language produced significant changes in facial expressions. Therefore, biographical music has the potential to evoke physiological and neurophysiological effects in people with DOC.

Music can also be valuable as a diagnostic element for patients with DOC (38). Magee et al.'s (39, 40) *Music Therapy Assessment Tool for Awareness in Disorders of Consciousness* (MATADOC) combines music therapeutic and diagnostic strategies based on research findings suggesting enhanced auditory responsiveness in patients with DOC (41–43). The 14 items of MATADOC contain visual and auditory stimuli (39). These stimuli are used to evaluate motor responses and non-verbal communicative signs. Most of the stimuli consist of music, such as familiar songs. An evaluation of the MATADOC showed that this diagnostic tool is as reliable as other diagnostics in assessing behavioral signs of consciousness (39, 44).

In summary, music interventions that include biographic music and music therapy can lead to favorable responses in people with DOC. To this date, there seems to be no study that examined the impact of music on the endocrine system of people with DOC.

### Hormones and Music

Listening to music and creating music have been shown to have an impact on the endocrine system (45). Listening to music may reduce cortisol concentration in the saliva and increase the secretory immunoglobulin A [IgA; (46)]. Cortisol, a steroid that is produced by the hypothalamus-pituitary-adrenal (HPA) axis, is secreted in reaction to stress and anxiety (47, 48). Similar results were found by Linneman et al. (49). They discovered that music listening evoked a significant decrease in the cortisol concentration in saliva. The researchers measured the concentration of salivary alpha-amylase (SAA), an enzyme that breaks down carbohydrates (50). SAA concentration is an indicator of sympathetic nervous system activity (51). Linneman et al. (49) found that the change in SAA concentration was dependent on the qualities of the music being played. For instance, energetic music increased the concentration of this biomarker whereas relaxing music decreased the concentration. In a larger, randomized controlled study, 60 participants were first exposed to relaxing music, the sound of rippling water, or silence, and then to a stressful task (52). The cortisol concentration in the group, which listened to relaxing music, was highest, and the group, which listened to rippling water, had the lowest cortisol level. Alpha-amylase was decreased after the stress task more in the music group and in the silence group than in the group, which listened to rippling water. HR response showed no significant difference between the three groups (52). Kreutz (53) examined the impact of music making on different hormones. About 30 min of singing in a choir increased the concentration of oxytocin significantly, whereas this was not the case for cortisol and dehydroepiandrosterone [DHEA; (53)]. DHEA is a hormone that is produced by the HPA axis (54) and its level increases with relaxation (55, 56). Psychological stress (57) and depression (58) are associated with decreased DHEA levels. Therefore, DHEA decreases and increases opposite of cortisol and it can be an indicator for well-being.

In another study, 25 people with dementia received eight sessions of music therapy of 60 min within a 2-month period (59). Before and after the sessions, salivary IgA and alpha-amylase were collected and the participants had to fill in a mood questionnaire. The concentration of IgA and alpha-amylase was increased after music therapy, but the results were not statistically significant. Happiness scores, however, were found to significantly increase after music therapy interventions.

### Aims and Hypotheses

To our knowledge, the relationship between hormones and music in people with UWS and MCS has not yet been investigated. The purposes of this explorative study were (1) to examine the impact of biographical music and biographical language on physiological responses and (2) to examine the impact of biographical music and biographical language on the endocrine system of people with DOC.

We hypothesized that biographical music and biographical language evoke more bodily reactions than silence (H1). We also assumed that the physiological responses were greater in the music condition than in the language condition (H2). The following hormones were included in the study: salivary cortisol, amylase, and DHEA. The following physiological parameters were included in the study: electromyography (EMG), blood volume pulse (BVP), SpO_2_, and HR.

## Materials and Methods

### Participants

Twenty-four participants with UWS and MCS were recruited at two neurological early rehabilitation departments (Department of Neurological Intensive Care and Rehabilitation, Evangelisches Krankenhaus Oldenburg and Clinic of Neurological Rehabilitation, Asklepios Schlossberg Klinik Bad König) and one long-term rehabilitation facility (Nursing Care Facility Schloss Meerholz, Area Phase F, Part A, Gelnhausen). Inclusion criteria were an age of 18 and older, an absence of dementia, no prior drug or alcohol abuse, no hearing deficit, no mechanical ventilation, and an absence of multidrug-resistant organisms in the area of the face, mouth, or tracheostomy. Between the study recruitment and study interventions, four participants were excluded due to multidrug-resistant germs (*n* = 2), meningitis (*n* = 1), or emergence from MCS (*n* = 1). Therefore, 10 men and 10 women [*M* = 55.1 years, standard deviation (*SD*) = 15.09] participated in the study. Fourteen participants lived with UWS and six with MCS (as shown in Table 1). Hypoxia (*n* = 9) was the most common etiology, followed by traumatic brain injury (TBI; *n* = 7), intracranial hemorrhage (ICH; *n* = 2), subarachnoid hemorrhage (SAH; *n* = 1), and tumor edema with herniation (*n* = 1). The injury's onset was 1.5 months to 30 years ago (*M* = 4.64 years, *Mdn* = 3.25 months). Three different types of assessments were conducted by physicians or trained therapists before the interventions: the *GCS* (11), the CRS-R (13), and the *Glasgow Outcome Scale* [GOS; (12)]^1^.

**Table 1:** Participants' characteristics.

**Patient no**.	**Age (y)**	**Etiology**	**GCS total**	**GCS subscores (E/V/M)**	**CRS-R total**	**CRS-R subscores (A/Vi/M/O/C/Ar)**	**GOS**	**Diagnosis**
1	50–55	TBI	4	2/1/1	4	1/0/1/1/0/1	2	UWS
2	65–70	TBI	5	3/1/1	8	2/2/1/1/0/2	3	MCS
3	55–60	Hypoxia	4	2/1/1	2	0/0/0/1/0/1	2	UWS
4	40–45	Hypoxia	9	4/2/3	5	0/0/1/2/0/2	2	UWS
5	70–75	Hypoxia	6	4/1/1	4	0/1/0/1/0/2	2	UWS
6	15–20	TBI	9	4/1/4	8	2/1/2/2/0/1	3	MCS
7	65–70	SAH	8	4/1/3	2	0/0/1/0/0/1	2	UWS
8	55–60	Tumor	6	2/1/3	2	0/0/1/0/0/1	2	UWS
9	75–80	TBI	8	3/1/4	7	2/1/2/1/0/1	3	MCS
10	55–60	Hypoxia	8	4/1/3	4	1/0/1/0/0/2	2	UWS
11	70–75	ICH	6	2/1/3	3	1/0/1/0/0/1	2	UWS
12	60–65	Hypoxia	10	4/3/3	8	2/1/1/2/0/2	3	MCS
13	30–35	TBI	8	4/1/3	7	2/1/1/1/0/2	3	MCS
14	55–60	Hypoxia	7	4/1/2	5	1/1/0/1/0/2	2	UWS
15	55–60	Hypoxia	7	4/1/2	5	1/1/0/1/0/2	2	UWS
16	55–60	Hypoxia	6	4/1/1	3	0/0/0/1/0/2	2	UWS
17	25–30	TBI	4	2/1/1	4	1/0/1/1/0/1	2	UWS
18	65–70	ICH	5	3/1/1	4	1/1/0/1/0/1	2	UWS
19	50–55	Hypoxia	4	2/1/1	2	0/0/0/1/0/1	2	UWS
20	45–50	TBI	7	4/2/1	8	1/2/1/2/0/2	3	MCS

*TBI, traumatic brain injury; ICH, intracranial hemorrhage; SAH, subarachnoid hemorrhage; y, years; GCS, Glasgow Coma Scale; E, eye opening; V, verbal response; M, motor response; CRS-R, Coma Recovery Scale Revised; A, auditory function; Vi, visual function; O, oromotor/verbal function; C, communication; Ar, arousal scale; GOS, Glasgow Outcome Scale; UWS, unresponsive wakefulness syndrome; MCS, minimally consciousness state*.

### Ethics

The study was approved by the Ethics Committee of the Carl von Ossietzky University, Oldenburg [number 115/2016 (075/2016)] and was designed consistent with the principles of the Declaration of Helsinki (60). The legal representatives signed informed consent. The study is reported in the German register of clinical trials (https://www.drks.de/drks_web/setLocale_EN.do; clinical trial identifier number DRKS00010187). Each participant was anonymized to a four-digit pseudonym consisting of random numbers and letters.

### Music and Language Stimuli

The legal representatives completed a questionnaire about preferred biographical music and language. This information was used to create individual stimuli for each participant. The biographical language stimuli consisted of audiobooks, television broadcasts, books, or newspaper articles. In cases of books or newspaper articles, the text was read out by an actor and recorded. For the music stimuli, four preferred songs were chosen. In one case, the family of a participant provided a record of a choir, in which the participant sang. The samples were edited with a program for audio editing (Audacity 2.3; iWeb Media Ltd., Birkirka, Malta). They began with a fade-in of 5 s and ended with a fade out of 5 s. Between the songs or language pieces, a break of 1 min silence was inserted.

### Design and Procedure

Prior to the start of the study, a power analysis was conducted with the program G^*^Power (61) to determine the appropriate study size. For this purpose, a two-factor analysis of variance (ANOVA) with repeated measures was utilized. The two factors represented the biographical language and biographical music of independent variables. The analysis led to a sample size of 20 participants, a significance level of α = 0.5, a power of β = 80%, and an effect size of 0.28.

**Figure 1:**
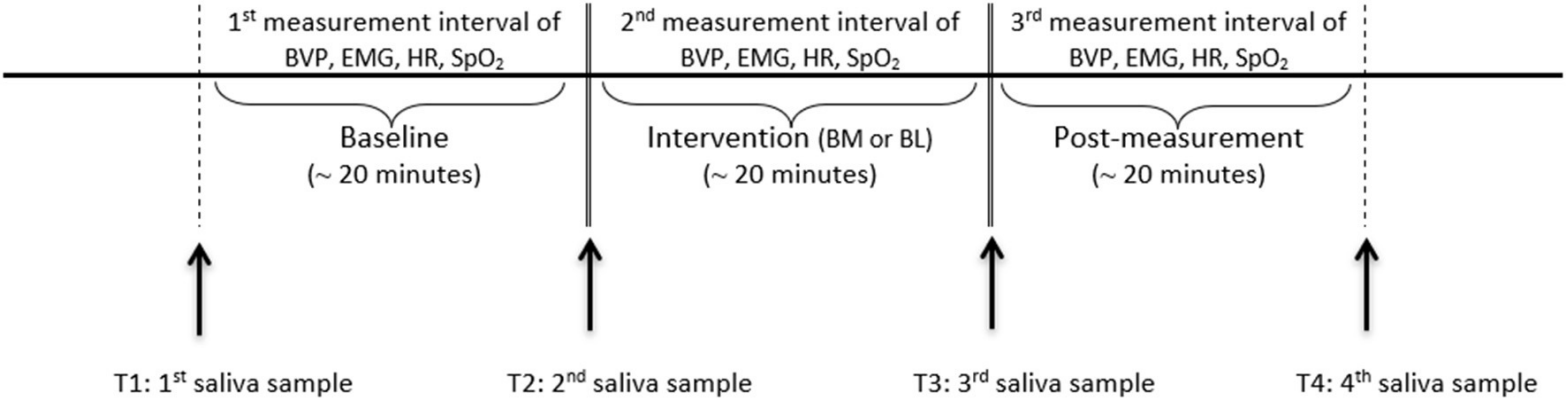
Experimental design. BM, biographical music; BL, biographical language.

The study took place at the bedside. The patients participated on 2 consecutive days at the same time in the music condition and in the language condition. The interventions took place at the same time as cortisol and alpha-amylase levels fluctuate with circadian cycles (47, 51). Music and language followed for each participant in a randomized order. The two conditions were assigned by drawing lots. Due to this randomization, some participants started with music and some with language on the first day. The interventions lasted approximately 20 min per condition. Before and after the interventions, baseline and post-intervention measurements were obtained for 20 min (as shown in Figure 1).

### Data Acquisition

Data collection was performed between September 2017 and September 2019. *The NeXus-10 MKII* (62) was utilized to measure the physiological parameters. This device complies with the International Electrotechnical Commission (IEC) 60601 standard and is approved as a medical electronic device. The parameters were acquired continuously during the baseline, the intervention, and the post-measurement (as shown in Figure 1). HR and BVP were measured by a finger clip at the index finger of the non-dominant hand. SpO_2_ was measured by a finger clip at the index finger of the dominant hand. The electrodes for the EMG were placed at the forehead at the frontalis muscle and a ground pole above the root of the nose. Saliva was collected four times per intervention. The first saliva sample was collected before the baseline (T1), the second (T2) and the third one (T3) before and after the intervention, and the last one after the post-measurement (T4). A Salivette^©^ Cortisol (Sarstedt, Nümbrecht, Germany) with a synthetic fiber swab was used for this procedure. The biocompatible fiber roll was placed in the cheek pouches for 5 min and held tight by the experimenter. The sample was then stored in a sterile tube at −20°C.

During the interventions, the room temperature was maintained between 20 and 24°C. The loudspeakers for the preferred music and language were placed at the foot side in front of the bed. The distance between the heads of the participants and the loudspeakers was maintained at 2.50 m. The volume was controlled by the sound level meter *Testo 816* (Testo, Titisee-Neustadt, Germany) next to the participant's head and was held between 60 and 65 dB. The sound level meter is approved for the frequency range from 31.5 to 8 kHz. The accuracy of the device corresponds to class 2 according to IEC 651 (DIN EN 60651) and is ±1.0 dB. Finally, a protocol was written in which confounders or special reactions were noted by the examiner.

### Data Processing and Analyses

The physiological data were analyzed with *BioTrace*+ (63). Artifact rejection was conducted to eliminate confounders, for example, when an electrode fell down and had to be placed at the participant again. First, the artifacts that were noted in a protocol (e.g., background noise, a loose electrode) were eliminated. In a second step, two neurologists, who were blinded to the conditions, eliminated biological and technical artifacts—such as movement artifacts—by using visual inspection. Then, the data were exported to the statistical program *IBM-SPSS Statistics* (version 26, New York, NY, USA). The mean value of the three time intervals (baseline, interventions, post-measurement) was used for statistical analysis. ANOVAs with repeated measurements and two factors were used (α = 0.5, β = 80%). The two factors represented biographical music and biographical language. The ANOVA's repetitions represented the measurement times (baseline, intervention, and post-measurement; T1–T3). Additionally, significance was explored based on the Student's *t*-test for dependent measures (α = 0.5, β = 80%).

The saliva samples were cooled and transported with dry ice to a lab. The lab was blinded to the participants and conditions. Free cortisol concentration and DHEA concentration were determined by using commercial luminescence immunosorbent assay (LUM) and commercial enzyme-linked immunosorbent assay (ELISA) from the same manufacturer (IBL International, a Tecan Group company, Hamburg, Germany), respectively. The SAA activity was measured using a kinetic colorimetric test [for details, refer to (64)] and reagents obtained from DiaSys Diagnostic Systems (Holzheim, Germany). Saliva was diluted at 1:400 using 0.9% saline solution. The reagents contained the enzyme alpha-amylase in a specified amount and alpha glucosidase, which converts the substrate ethylidene nitrophenyl to p-nitrophenol. The rate of formation of p-nitrophenol is directly proportional to the samples' amylase activity and was detected using an absorbance reader at 405 nm (BioTek Synergy HTX, BioTek Instruments, Winooski, VA, USA). Inter-and intra-assay coefficients of variation were below 10% for all analytes.

A two-factor ANOVA with repeated measurements was used for statistical analysis (α = 0.5, β = 80%). The ANOVA's repetitions represented the times at which the saliva was acquired (T1–T4). In addition, the mean values of the four measurement times were compared by applying the Student's *t*-test for dependent measures (α = 0.5, β = 80%).

## Results

### Psychophysiological Data

The ANOVAs and the Student's *t*-tests for paired samples showed no significant changes due to biographical music or biographical language in BVP, EMG, SpO_2_, and HR (as shown in Table 2).

**Table 2:** Means (and standard deviations) of physiological responses to biographical music and language stimulation across time points.

	**T1**	**T2**	**T3**
	**M (SD)**	**M (SD)**	**M (SD)**
**BVP**			
Music	−42.17 (71.17)	−29.97 (16.12)	−29.38 (17.23)
Language	−52.76 (79.79)	−41.05 (36.59)	−35.59 (23.11)
**EMG raw**			
Music	3510.65 (6743.43)	3671.60 (6,481.06)	3327.80 (6081.54)
Language	3538.20 (6240.71)	3428.29 (4090.20)	3186.48 (3310.30)
**EMG (20–500 Hz)**			
Music	0.0086 (0.1128)	−0.1209 (0.544)	0.0043 (0.0374)
Language	0.0263 (0.0791)	0.0479 (0.36)	0.0803 (0.3819)
**HR**			
Music	77.59 (14.44)	78.63 (14.49)	77.77 (14.32)
Language	75.80 (12.65)	73.96 (13.67)	74.49 (13.92)
**SpO**_**2**_			
Music	93.88 (2.89)	93.72 (2.98)	93.60 (2.88)
Language	94.06 (2.85)	93.87 (2.86)	94.10 (2.62)

*BVP, blood volume pulse, relative blood flow in millivolts; EMG, electromyography in microvolts (μV pk-pk); HR, heart rate in beats per minute; SpO_2_, oxygen saturation measured by a finger pulse oximeter in percent*.

### Hormones

**Figure 2:**
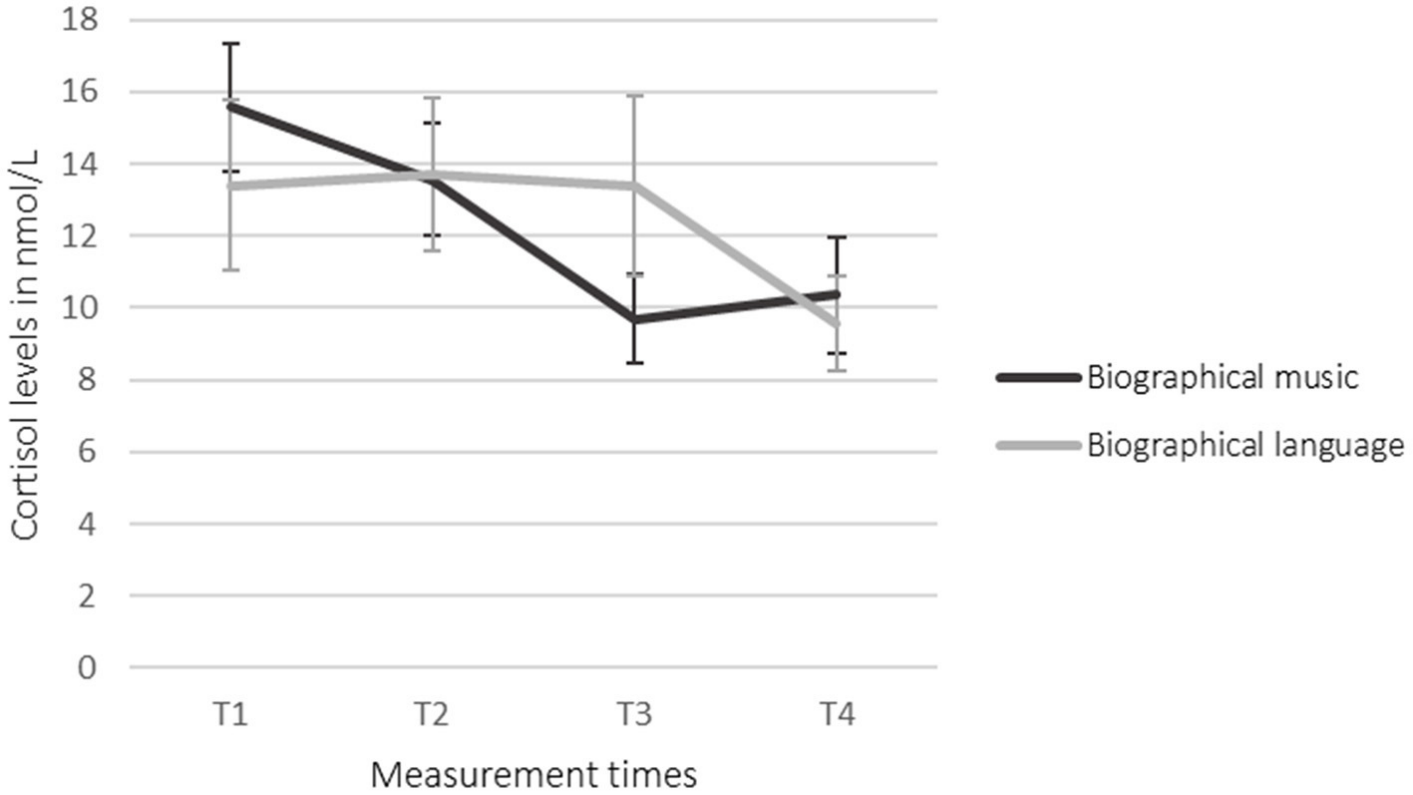
Change in cortisol levels. Error bars represent the standard error of the mean (SEM).

The ANOVAs showed no statistically significant effects. The Student's *t*-tests for paired samples showed that cortisol in saliva was decreased significantly in the language condition (T2–T4) {[95% CI (0.998, 7.278)] *t*_(19)_ = 2.758, *p* = 0.013, *d* = −0.536}. Between T3 and T4 in the language condition, the salivary cortisol was decreased significantly, as well {[95% CI (0.198, 6.989)] *t*_(18)_ = 2.223, *p* = 0.039, *d* = −0.437}. Biographical music led to no significant effects. Salivary cortisol was decreased after the intervention (T3) in comparison to the baseline (T2) (as shown in Table 3; Figure 2). Biographical music and biographical language led to no significant changes in salivary amylase and DHEA.

**Table 3:** Means (and standard deviations) of neurohumoral responses to biographical music and language stimulation across time points.

	**T1**	**T2**	**T3**	**T4**
	**M (SD)**	**M (SD)**	**M (SD)**	**M (SD)**
**Amylase^a^**				
Music	293.07 (340.48)	320.14 (395.88)	280.61 (291.88)	237.30 (246.42)
Language	205.46 (151.00)	393.46 (617.09)	336.40 (418.21)	348.39 (392.85)
**Cortisol^b^**				
Music	15.58 (16.36)	13.55 (17.32)	9.70 (5.28)	10.36 (6.85)
Language	13.41 (9.77)	13.70 (9.13)	13.38 (10.83)	9.56 (5.97)
**DHEA^c^**				
Music	188.94 (173.29)	170.58 (135.94)	176.80 (128.15)	165.84 (141.69)
Language	179.34 (141.54)	184.08 (144.60)	228.22 (277.33)	157.57 (113.77)

a *sAA in U/mL*.

b *sCort in nmol/L*.

c *DHEA in pg/ml*.

*DHEA, dehydroepiandrosterone*.

## Discussion

In this study, 20 participants received biographical interventions. In reaction to biographical music, salivary amylase did not decrease significantly after the intervention in comparison to the baseline (T2 to T3) and from T3 to T4. In reaction to biographical language, salivary amylase also did not appear to significantly change. Consequently, biographical interventions had no significant effects on the production and release of salivary amylase.

Biographical music had no effect on the concentration of DHEA in saliva, which stayed at the same level during the baseline, intervention, and post-measurement. Biographical language elicited a non-significant change from T2 to T3. The SD in DHEA was in comparison to the mean value very high. An explanation for this may be that the level of DHEA decreases with age (65, 66). DHEA levels have been found to be significantly decreased when comparing people aged 50 to those aged 40 years old (65). The age of the participants included in our study was very heterogeneous with a range between 19 and 77 years. Similarly, the level of amylase is also influenced by age (50). The level of cortisol is influenced by age, but it is less affected by age than levels of DHEA (65).

Although the mean values suggested a decrease of salivary cortisol levels in the participants' saliva during listening to biographical music (T2 to T3) followed by an increase in levels after the intervention (T3 to T4), the changes were not statistically significant. During the language intervention, salivary cortisol was decreased from T2 to T4 and from T3 to T4 significantly with *p* < 0.05. This could be due to a delayed reaction. It remains unclear if this effect is in reaction to the intervention's content, the voice of the reader, or the sound of the audiobooks. Since the participants had a severe brain injury, most of them might have aphasia (4, 67) preventing them from understanding the content of the language stimuli. The decrease of cortisol in the language condition could therefore be due to the sensitivity to prosodic features of the language. Prosodic features of the native language are perceived by babies in an early stage of language development: Infants who are 6 months old are able to discriminate between the native prosody and the prosody of a language that is prosodically very different from their native language (68). This sensitivity occurs even before children are able to understand the semantic content of a language and produce the first word (69). Emotional prosody, which is not a linguistic feature but contains social information, is processed by babies from the age of 6 months onward (70, 71).

Cortisol was found to be more meaningful than amylase and DHEA in this study. Cortisol may be released in reaction to stress (72) and therefore, this can be an indication that the interventions had a calming effect. The role of cortisol differs in other studies with participants with brain injuries. Kleindienst et al. (73) measured cortisol levels in the blood of 71 participants with TBI in the acute phase. The blood samples were taken between 7:00 and 9:00 a.m. on days 0, 3, and 7. On the seventh day, a 24-h urine sample was taken. Patients with a moderate brain injury had a significantly higher cortisol level in the blood than patients with severe TBIs. However, in the 24-h urine sample, increased cortisol levels were found. Heinz and Rollnik (35) compared cortisol levels of patients with a poor outcome and patients with a good outcome in 93 patients with hypoxia. The mean duration of the treatment in the rehabilitation unit was 108.5 days. The results showed that there was no significant difference in cortisol levels in patients with a poor outcome (193.00 μg/l) and patients with a good outcome (194.00 μg/l). Vogel et al. (74) explored the circadian rhythm of cortisol in patients with UWS who were in the subacute and chronic rehabilitation phase. For this purpose, they measured the 24-h profiles of 11 patients with UWS and a neurologically healthy control group that consisted of 11 age and gender-matched participants. The circadian rhythm was preserved in all patients but it differed from the control group. The 24-h mean of cortisol was higher in patients than in the neurologically healthy participants. Between 16:00 p.m. and 20:00 p.m., the patients' cortisol levels were higher than those of the control group. Consequently, the role of cortisol in people with brain injuries is contradictory and might depend on the rehabilitation phase and etiology. However, the present study showed that biographical language can induce a decrease in cortisol levels in people with UWS and MCS in the subacute and chronic rehabilitation phases.

The results of this study are partly in line with the study by Puggina and da Silva (37) mentioned previously. In Puggina and da Silva's study, the participants listened to the voice of a relative in the language condition and showed significant behavioral reactions. The familiarity of the voice might be the reason for the participants' strong reactions.

In comparison to a study by Heine et al. (42), biographical language led to more physiological reactions than biographical music in our study. Heine et al. (42) used preferred music that was reported by the participants' families or loved ones. They found out that the preferred music evoked more functional connectivity in the left precentral gyrus and the left dorsolateral prefrontal cortex than in the control condition (noise from the MRI scanner). Despite the small number of five participants, the intervention showed effects of preferred music on brain regions that process cognitive functions, such as the dorsolateral prefrontal cortex (42). Moreover, the right and left gyrus precentralis showed significantly more connectivity in the music condition when compared to the control condition. Therefore, biographical music may evoke higher neural connectivity. A follow-up study conducted by the same team (25) supported these results by showing more connectivity in the frontoparietal network during the music intervention than in the control condition.

Castro et al. (75) also worked with biographical music. They presented four iterations of preferred music excerpts for 1 min to 13 participants with DOC. The control condition consisted of a continuous sound. The participants listened to these two conditions alternately. After the two conditions, an EEG measurement was conducted and during that, the participants listened to different prenames, which included their own names. The EEG response to participants' own names was higher after music interventions than after the control condition. Seven patients showed a significantly higher response to their own names in comparison to other names after they listened to music. These seven participants had a better outcome after 6 months. The remaining six participants did not show such reactions. Even though the number of participants was low, the study showed that biographical music induced cognitive processes in half of the participants.

The results of the present study and the results of previous studies suggest that biographical interventions affect emotional and cognitive function in some people with DOC. As people with DOC cannot express themselves verbally, there will be no full certainty about how they perceive therapeutical interventions. Johnson (76) points out that such circumstances should not preclude further attempts in research or treatment that are made to enhance the quality of life for people with DOC. She calls this “ethics of uncertainty” [(76), p. 190]. Therefore, professionals have to deal with uncertainty when working with people with DOC in a rehabilitative or therapeutic setting.

### Limitations

The present study was not fully blinded, as the examiner both collected and analyzed the data. Some of the participants were more in the acute state and some in a chronic state. Thus, the participants' conditions were heterogeneous. The stimuli differed in the type of genre, structure, instruments, and other features. Previous research showed that different (structural) features of music influence physiological reactions, for example, in HR, or respiratory rate (RR) in healthy populations (77, 78), and HR, heart rate variability (HRV), RR, or SpO_2_ in people with DOC (79, 80).

## Conclusion

The findings of this study are in line with previous research and add some new aspects by exploring hormones. Biographical language led to a significant decrease in salivary cortisol. This suggests that biographical interventions might reduce stress in people with UWS and MCS. The age of the participants and the time since their brain injuries, however, were heterogeneous. Therefore, the results are not generalizable. More research is needed to investigate the effects of different arts-based interventions and responses of the endocrine system in people with DOC.

## Data Availability Statement

The data supporting the conclusions of this article will be made available by the authors

## Ethics Statement

The studies involving human participants were reviewed and approved by Medical Ethics Committee, Carl von Ossietzky University, Oldenburg, Germany. The participants' legal representatives provided written informed consent to participate in this study.

## Author Contributions

TG and GK conceptualized the study. TG acquired the data and wrote the first draft of the manuscript. TG, MG, and OS analyzed the data. UN provided the analysis of salivary biomarkers at his lab. All authors contributed to writing the manuscript and approved the submitted version.

## Conflict of Interest

The authors declare that the research was conducted in the absence of any commercial or financial relationships that could be construed as a potential conflict of interest.

## Publisher's Note

All claims expressed in this article are solely those of the authors and do not necessarily represent those of their affiliated organizations, or those of the publisher, the editors and the reviewers. Any product that may be evaluated in this article, or claim that may be made by its manufacturer, is not guaranteed or endorsed by the publisher.

## Footnotes

^1^The diagnosis of UWS or MCS is based on the best result of the three assessments.
